# Health-related quality of life of people with depression: pre-post intervention compared with age-matched general population in Vietnam

**DOI:** 10.1186/s40359-024-02067-z

**Published:** 2024-10-17

**Authors:** Truong Thi My Hoa, Tran Thu Ngan, Vu Quynh Mai, Hoang Van Minh, Nguyen Khac Thu, Tran Kieu Nhu

**Affiliations:** 1 Caring From Distance Center for Research and Community Development, Hanoi, Vietnam; 2 Centre for Public Health, Queen’s University Belfast, Belfast, United Kingdom; 3 Centre for Population Health Sciences, Hanoi University of Public Health, Hanoi, Vietnam; 4 Institute for Social Development Studies, Hanoi, Vietnam

**Keywords:** Health-related quality of life, Depression, Supported self-management, Task-shifting approach, Mobile health, Mental health

## Abstract

**Background:**

In Vietnam, there is a paucity of health-related quality of life (HRQoL) research on people with depression as well as a lack of evidence on supported self-management (SSM) intervention for depression on HRQoL. This study aimed to compare the HRQoL of people with depression and age-matched people in the Vietnamese population, evaluate the effects of Tele-SSM intervention on HRQoL, and examine the association between the changes in HRQoL score and mental health well-being (depression, anxiety, and stress).

**Methods:**

This study was a pre- and post-study involving Vietnamese adults aged 18–64 who had depression symptoms (score ≥ 5 points in the PHQ-9). Participants (*N* = 58) completed Tele-SSM—a supported self-management intervention incorporating cognitive behavioral therapy and non-violent communication and using a task-shifting approach. To compare with the general population, we conducted a 1-to-1 matching by age with an EQ-5D-5L valuation study with a nationally representative sample of the Vietnamese general population.

**Results:**

HRQoL was significantly impaired in people with depression compared to the age-matched general population. Regarding health profile, participants reported the most difficulties in usual activities, pain/discomfort, and anxiety/depression dimensions. Significant improvements were observed in HRQoL scores post-intervention compared to pre-intervention in both EQ-VAS scores (from 59.84 to 79.48) and utility scores (from 0.79 to 0.90). These results remained lower than the age-matched general population (EQ-VAS score = 83.28, utility score = 0.96). Depression was associated with lower HRQoL while controlling for stress, anxiety symptoms and sociodemographic characteristics.

**Conclusions:**

These results indicate that HRQoL is significantly impaired in people with depression and contribute to providing empirical evidence of Tele-SSM intervention in improving HRQoL. Further randomized controlled trials should be conducted to evaluate the effectiveness and cost-effectiveness of the Tele-SSM intervention.

## Background

The Global Burden of Diseases study found that depression affected 280 million people around the world in 2019, including an estimated 5% of adults and 1.8% of disability-adjusted life years [12, 13]. Depression is also among the leading causes of disability, which increases the burden of disorder globally [48]. A systematic review predicted the prevalence of depression still increase as a result of the COVID-19 pandemic, even estimated to rank 2nd among the leading causes of burden of disease in 2030 [31, 32, 42]. In Vietnam, studies reported the prevalence of depression among adults and older people was about 12.7% and 26.4%, respectively [9, 49].

Previous studies consistently found health-related quality of life (HRQoL) impairment in people with depression [8,21,22,44]. Despite of being improved after completing the acute treatment phase, the HRQoL of the majority of patients whose depression remitted was still lower than the general population [1, 22]. In Vietnam, the impact of depression on HRQoL was documented in a few studies with HIV/AIDS, lung cancer patients, and the elderly only, and found that depression has been negatively associated with HRQoL in these populations [24, 45, 52]. To our knowledge, there are no published papers investigating HRQoL of people with depression in Vietnam.

Literature reveals a robust body of evidence on the effects of psychotherapy for depression on HRQoL. A meta-analysis exploring the effect of psychotherapy for depression showed that psychotherapy for depression has a positive impact on HRQoL with a small to moderate effect size (Hedges’ g = 0.33) [25]. In another meta-analysis, cognitive behavioral therapy (CBT) had a moderate size effect (Hedges’ g = 0.63) in improving HRQoL for adults with depression and the effect remained stable over the follow-up stage [19]. Some studies found that there are no significant differences in the effect of psychotherapy for depression on HRQoL between telehealth delivery and face-to-face delivery [4, 10]. Telemental health offers several key advantages, including accessibility, lower costs, and greater convenience while maintaining interaction between patients and providers. This is especially valuable in regions with limited mental health resources [26].

Supported self-management (SSM) for depression is an intervention that teaches participants knowledge and skills for coping with depression through self-management guides (workbook, website) based on CBT principles [2]. This program is recommended by The UK National Institute for Health and Care Excellence (NICE) guideline as guided self-help for mild and moderate depression [36]. In Vietnam, a randomized controlled trial of an SSM intervention for depression in a community-based setting showed a reduction in depression symptoms [34]. Our team conducted a study on the primary efficacy, feasibility, and acceptability of Tele-SSM intervention which is SSM intervention delivered from a distance. Findings showed that participants after the Tele-SSM intervention experienced significantly reduced symptoms of depression, anxiety, stress, and suicidal ideation compared to the baseline (Tran NK, Ngo TT, Nguyen TK, Murphy J, Dang MH: Preliminary efficacy, feasibility and acceptability of a telehealth supported self-management intervention for adults with depression symptoms in Vietnam, unpublished). Regarding the effect of SSM intervention on HRQoL, there is still limited empirical evidence. A few studies conducted in high-income countries found that SSM significantly improved HRQoL [16, 47].

In the context of limited knowledge about HRQoL of people with depression in Vietnam as well as the effect of SSM intervention on HRQoL in literature, especially in a limited resources context, the current study aims to (1) assess HRQoL of people with depression in comparison with age-matched general population; (2) evaluate the effect of Tele-SSM intervention on HRQoL; (3) examine the association between the changes in HRQoL score after intervention and mental health wellbeing (depression, anxiety, and stress), controlling by sociodemographic characteristics.

## Methods

The Tele-SSM study was conducted from April 2021 through August 2023 in Vietnam by the Institute for Social Development Studies (ISDS). It was approved by the Institutional Review Board at ISDS (Ref No. IRB00011703) in Hanoi. Informed consent was obtained from all participants in the study.

### Study design

Tele-SSM is a pre-post mixed-methods study of which the quantitative component was used in this paper. Each participant completed an online survey hosted on KoboToolbox platform at baseline and at the end of the intervention. The survey included sociodemographic information, knowledge-attitude-practice (KAP) regarding depression, psychosocial well-being, and HRQoL.

### Participants and recruitment

The inclusion criteria for participants were 18–64 years old with depression symptoms (i.e., score 5 points or above in the Patient Health Questionnaire (PHQ-9)). The exclusion criteria were having severe mental health problems (i.e. schizophrenia, bipolar, and suicide attempts). Participants were recruited through social media (organization website, Facebook). Potential participants completed a screening questionnaire on Kobotoolbox. Those meeting the study criteria based on the online questionnaire were contacted for a video interview by a research assistant with a psychology background to confirm eligibility, provide study details, and obtain informed consent. Eligible participants received the printed intervention manual and workbook through postal mail. 75 participants were enrolled in the study and 58 completed the intervention. The average age of participants was 33.14 ± 8.87. The majority (98.3%) belonged to the Kinh ethnicity, and 89.7% of participants were female and resided in large town or city. Most participants (82.8%) were employed, with 87.9% having an education level from college and above. Regarding living situations, 82.8% lived in family households, 12.1% lived alone, and 5.2% lived with friends (Table 1).

**Table 1:** Sociodemographic characteristics of participants (*N* = 58)

	Tele-SSM participants (*n* = 58)
Characteristic	n (%) or Mean (SD)
Age, years (SD)	33.14 (8.87)
Male, n (%)	6 (10.3%)
Kinh majority ethnicity, n (%)	57 (98.3%)
Place of residence	
Rural area or small town	6 (10.3%)
Large town or city	52 (89.7%)
Living situation, n (%)	
Alone	7 (12.1%)
In a family household	48 (82.8%)
With one or more friends	3 (5.2%)
Employment situation	
Employed	48 (82.8%)
Unemployed	10 (17.2%)
Student	0 (0%)
Highest level of education achieved	
Highschool	7 (12.1%)
College/university	47 (81%)
Master/PhD	4 (6.9%)

The validation study of the EQ-5D-5L questionnaire in Vietnam employed a nationally representative sample of 1200 adults, recruited through multi-stage stratified cluster sampling across six provinces. Participants were selected using quota-based sampling to reflect age and gender demographics [30].

### Tele-SSM intervention

Tele-SSM is a supported self-management intervention for depression delivered over phones. It was developed and modified based on the Live Happily intervention—a psychosocial telehealth intervention to address multi-level stigma among youth living with HIV in Vietnam. Live Happily was developed based on a face-to-face supported self-management for depression intervention [33, 34] (Tran NK, Ngo TT, Nguyen TK, Murphy J, Dang MH: Preliminary efficacy, feasibility and acceptability of a telehealth supported self-management intervention for adults with depression symptoms in Vietnam, unpublished). Live Happily decreased depression symptoms significantly for youth living with HIV. Tele-SSM was further adapted to address depression more specifically by incorporating additional psychoeducation and behavior activation skills. The intervention manual was developed by the principal investigator based on the Live Happily manual and refined from expert feedback. The feedback was collected from five experts in psychology, psychiatry and community-based mental health care. Before implementation, the updated manual was tested with 10 individuals with depression.

The Tele-SSM intervention incorporates CBT and non-violent communication and uses a task-shifting approach, which is an approach where non-specialist providers deliver evidence-based, cost-effective health services, redistributing tasks from highly trained professionals to less specialized workers to optimize healthcare resource utilization. The intervention consists of 10 weekly sessions (60 min each session) delivered via Zoom by a coach (audio only). Coaches were para-professionals with health, public health, social sciences, or education backgrounds, who were trained and supervised.

The topics of the 10 sessions are:

**Table:** 

Session 1: Depression psychoeducation & behavior activation Session 2: Relaxation skill Session 3: Realistic thinking Session 4: Nonviolent communication, part 1 Session 5: Nonviolent communication, part 2	Session 6: Problem-solving Session 7: Dealing with major events Session 8: Stepping out of comfort zone Session 9: Dealing with anger Session 10: The road ahead

### Measurements

#### Health-related quality of life

HRQoL was measured by the EQ-5D-5L questionnaire which was validated in Vietnam [30]. It consists of 2 scales: the EQ-5D scale and the EQ visual analogue scale (EQ VAS). The EQ-5D scale measures the level of difficulties in five dimensions: mobility, self-care, usual activities, pain/discomfort, and anxiety/depression according to 5-point Likert levels (no problems, slight problems, moderate problems, severe problems, unable to/extreme problems).

The EQ VAS records self-reported health on a vertical analogue scale from 0 (the worst health you can imagine) to 100 (the best health you can imagine).

#### Mental well-being

Depression was measured by the PHQ-9 [29] which was validated in Vietnam [35, 39]. The PHQ-9 demonstrated consistently high reliability in assessing depression across multiple Vietnamese studies. In our sample, the reliability is good with Cronbach’s α = 0.80.

Anxiety and stress were measured with the 21-item Depression, Anxiety and Stress Scale (DASS-21) [18, 28], which was validated in Vietnam [27, 46]. Our study showed excellent internal consistency for both the stress and anxiety subscales (Cronbach’s α = 0.90).

### Statistical analysis

The EQ-5D-5L questionnaire was analyzed to derive 3 sets of variables: 1) frequencies and proportions of participants’ reported levels of problems by five dimensions of health; 2) mean of EQ-VAS score; 3) mean of utility score. The utility score was calculated by deducting the corresponding weights of the five dimensions from 1 (full health). The weights for the EQ-5D scale were validated and calculated specifically for the Vietnam population [30].

To compare HRQoL between people with depression and the general population, we used the raw data from an EQ-5D-5L valuation study with a nationally representative sample of the Vietnamese general population [30]. We conducted 1-to-1 matching by age between participants in the Tele-SSM study and their peers in the general population (case and control, respectively). The matching procedure followed 3 steps: (1) data was randomly sorted,(2) the case was matched with the nearest control who had the same age or the age difference was less than a quarter of the standard deviation of age (recommended by [15]); (3) the matched pair or the unmatched case were removed from the dataset. These 3 steps were repeated until all the cases were matched. After matching, we compared HRQoL of people with depression before and after Tele-SSM intervention with age-matched controls: 1) EQ-5D frequencies and proportions by five dimensions by Chi-square tests. The level of problems on each dimension was dichotomized into ‘0 = no problems’ and ‘1 = any problems’; 2) mean EQ-VAS score and utility score by Mann–Whitney U tests as these variables were not normally distributed.

To evaluate the changes of HRQoL after the Tele-SSM intervention, we compared before and after the intervention: 1) EQ-5D frequencies and proportions by five dimensions by McNemar’s test; 2) mean of EQ-VAS score and utility score by Wilcoxon signed rank tests as these variables were not normally distributed. The effect size of Wilcoxon signed rank tests was calculated using the formula *r* = z / square root of N [37]. Effect size r was categorized into small, medium, or large according to Cohen [6] criteria of 0.1 = small effect, 0.3 = medium effect, 0.5 = large effect.

To examine the association between the changes in HRQoL score after intervention and mental health wellbeing, hierarchical linear regression was conducted with EQ-5D utility score and EQ-VAS score as outcome variables. The socioeconomic characteristics were added in the first step of the model. Depression, anxiety variables pre- and post-intervention were added in the second step of the model. We checked for multicollinearity between all variables in regression model and there was no multicollinearity between variables (including depression and anxiety).

## Results

### HRQoL of people with depression compared to aged-matched general population

#### Health profile across five health dimensions

Table 2 presents the health profile proportions of participants before and after the intervention and the age-matched general population who reported problems on each EQ-5D dimension. Compared with the age-matched general population, the proportions of people with depression having difficulties in usual activities, pain/discomfort, and anxiety/depression dimensions were significantly higher both before and after the intervention (all *p* < 0.05, Chi-square test).

**Table 2:** EQ-5D frequencies and proportions reported by dimension and groups of participants (*N* = 58)

	**General Population**	**Pre-intervention**		**Post-intervention**		***P*-value (Pre vs Post)**
	**n (%)**	**n (%)**	***p*-value (General vs Pre)**	**n (%)**	***p*-value (General vs Post)**	
**Mobility**						
No problems	54 (93.1)	49 (84.5)	0.141	56 (96.6)	0.675	0.039^*^
Slight problems	4 (6.9)	9 (15.5)		1 (1.7)		
Moderate problems				1 (1.7)		
Severe problems						
Unable/extreme problems						
**Self-care**						
No problems	58 (100)	58 (100)	-	57 (98.3)	1	1
Slight problems				1 (1.7)		
Moderate problems						
Severe problems						
Unable/extreme problems						
**Usual activities**						
No problems	58 (100)	32 (55.2)	< 0.01^**^	50 (86.2)	0.010*	< 0.001^**^
Slight problems		24 (41.4)		6 (10.3)		
Moderate problems		2 (3.4)		2 (3.4)		
Severe problems						
Unable/extreme problems						
**Pain/discomfort**						
No problems	42 (72.4)	16 (27.6)	< 0.01^**^	30 (51.7)	0.035*	0.004^**^
Slight problems	15 (25.9)	30 (51.7)		27 (46.6)		
Moderate problems	1 (1.7)	10 (17.2)		1 (1.7)		
Severe problems		2 (3.4)				
Unable/extreme problems						
**Anxiety/depression**						
No problems	49 (84.5)	2 (3.4)	< 0.01^**^	21 (36.2)	< 0.01^*^	< 0.001^**^
Slight problems	8 (13.8)	28 (48.3)		36 (62.1)		
Moderate problems	1 (1.7)	20 (34.5)		1 (1.7)		
Severe problems		7 (12.1)				
Unable/extreme problems		1 (1.7)				

The level of problems on each dimension were dichotomised into ‘0 = no problems’ and ‘1 = any problems’

^*^p < .05

^**^p < .01

#### EQ-VAS and utility scores (Figs. 1 and 2)

**Fig. 1:**
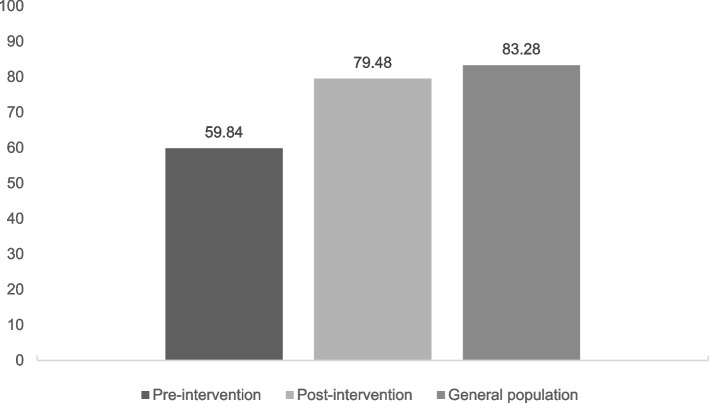
EQ-VAS in people with depression pre, post-intervention, and age-matched general population

**Fig. 2:**
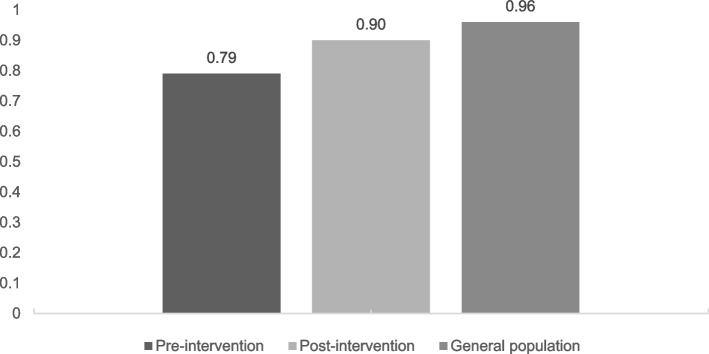
Utility scores in people with depression pre, post-intervention, and age-matched general population

Both the mean EQ-VAS and utility scores of people with depression before intervention were significantly lower than the scores of the age-matched general population (Mann–Whitney U tests, Z = -5.41, *p* < 0.01, and Z = -5.35, *p* < 0.01, respectively). After the intervention, only the mean utility score of people with depression was still significantly lower than the matched peers from the general population (Mann–Whitney U tests, Z = -5.41, *p* < 0.01); the mean EQ-VAS was not significantly different compared to the age-matched general population.

### Effect of Tele-SSM intervention on HRQoL

#### Health profile across five health dimensions

A significantly lower proportion of participants reported having problems in mobility, usual activities, pain/discomfort, and anxiety/depression dimensions after the intervention compared to those before the intervention (all *p* < 0.05, Chi-square test). There was no statistically significant difference between the proportions of participants having problems with self-care before and after the intervention.

#### EQ-VAS and utility scores (Figs. 1 and 2)

Regarding the changes in EQ-VAS and utility score of people with depression after the intervention, we found a significant increase in EQ-VAS and utility scores after participating in the intervention, with a large effect size for EQ-VAS score (*r* = 0.50) and a medium effect size for utility score (*r* = 0.49). The EQ-VAS mean score increased from 59.84 (SD = 17.39) pre-intervention to 79.48 (SD = 14.62) post-intervention and from 0.79 (SD = 0.10) to 0.90 (SD = 0.07) for utility mean scores (Figs. 1 and 2). In both cases, the increase was statistically significant (Wilcoxon Signed Rank Test, Z = -5.41, *p* < 0.001, and Z = -5.35, *p* < 0.001, respectively).

### Relationship between HRQoL changes and mental health outcome

#### Associations between variables: correlations

The correlations between background variables, sociodemographic characteristics, and outcome measures are presented in Table 3.

**Table 3:** Correlations between predictors and outcome variables

		**1**	**2**	**3**	**4**	**5**	**6**	**7**	**8**	**9**	**10**	**11**	**12**	**13**
1	Age													
2	Gender	-.04												
3	Education level	.47^**^	.05											
4	Personal income	.33^*^	-.29^*^	.18										
5	Depress pre-intervention	-.01	-.04	-.03	.11									
6	Stress pre-intervention	.06	.07	.11	-.03	.56^**^								
7	Anxiety pre-intervention	.03	.11	.00	-.12	.33^*^	.60^**^							
8	EQ-VAS score pre-intervention	.00	.13	-.07	.07	-.13	-.16	-.17						
9	Utility score pre-intervention	-.14	.19	-.01	.09	-.26	-.23	-.15	.49^**^					
10	Depress post-intervention	-.10	.19	-.02	-.05	.00	.02	.00	-.21	-.06				
11	Stress post-intervention	.02	.06	-.21	.09	-.01	-.14	-.20	-.10	-.04	.62^**^			
12	Anxiety post-intervention	-.07	.14	.17	-.13	-.19	-.18	-.05	-.16	-.06	.54^**^	.62^**^		
13	EQ-VAS score post-intervention	-.03	.11	-.03	.09	-.12	-.07	.09	.14	.15	-.49^**^	-.22	-.26	
14	Utility score post-intervention	-.09	-.08	-.10	.09	.28^*^	.26^*^	.07	.14	.20	-.51^**^	-.39^**^	-.46^**^	.27^*^

Gender (0 = Male, 1 = Female)

^*^p < .05

^**^p < .01

Depression, stress, and anxiety scores were significantly intercorrelated before and after the intervention indicating that there was comorbidity among mental health disorders.

EQ-VAS and utility score pre-intervention were positively related to each other. EQ-VAS score post-intervention was negatively associated with depression score post-intervention.

Utility score post-intervention was positively related to depression, stress pre-intervention and EQ-VAS score post-intervention while negatively associated with depression, stress, anxiety scores post-intervention.

#### Association between the changes in HRQoL score after intervention and mental health wellbeing

The findings of the regression analysis predicting EQ-VAS and utility scores found that controlling for sociodemographic characteristics (age, gender, education level, and personal income), higher depression score after intervention was associated with lower EQ-VAS and utility scores after intervention while no significant associations between anxiety/stress with EQ-VAS and utility scores were found after intervention (Table 4).

**Table 4:** Results of regression models predicting EQ-5D utility scores and EQ-VAS scores (*n* = 58)

**Step**			**EQ-VAS score *Adjusted R***^***2***^** = *.21***				**Utility score *Adjusted R***^***2***^** = *.30***		
			***Beta***		***p***		***Beta***		***p***
**1**	Age	-.25		.16		-.06		.71	
	Gender	.25		.10		-.01		.95	
	Education level	.17		.40		-.13		.48	
	Personal income	.17		.30		.06		.68	
**2**	EQ-VAS score pre-intervention	-.03		.82					
	Utility score pre-intervention					.25		.09	
	Depression pre-intervention	-.19		.26		.21		.21	
	Stress pre-intervention	-.14		.48		.30		.12	
	Anxiety pre-intervention	.29		.11		-.16		.36	
	Depression post-intervention	-.66^*^		< .00^**^		-.40*		.03^*^	
	Stress post-intervention	.40		.11		-.13		.58	
	Anxiety post-intervention	-.27		.24		-.40		.86	

Beta = coefficients beta of regression model

Gender (0 = male, 1 = female)

^*^p < 0.05

^**^p < 0.01

## Discussion

This study has a number of important findings. Firstly, people with depression reported significantly poorer HRQoL than the age-matched general population. Secondly, Tele-SSM improved the HRQoL of people with depression, but this remained lower than the age-matched general population, except for the EQ-VAS score. Thirdly, depression was associated with lower HRQoL while controlling for stress, anxiety symptoms, and sociodemographic characteristics.

Our findings indicated that people with depression had significant impairment in HRQoL in comparison with the age-matched general population. Our result is consistent with previous studies that people with depression were significantly more likely to have difficulties in usual activities, pain/discomfort, and anxiety/depression dimensions [14, 43]. The current study found that mean EQ-VAS and utility scores of people with depression in pre-intervention were significantly lower than age-matched peers in the general population. Regarding utility scores, our result is consistent with previous research that people with depressive disorder reported lower utility scores than the general population (0.73 vs 0.84, respectively) [41]. These results provide more empirical evidence of the impact of depression on HRQoL from limited resources context. In diversified socioeconomic circumstances, depression still impairs HRQoL significantly.

After completing the Tele-SSM intervention, participants showed great improvement in almost all dimensions, with a large effect size for EQ-VAS score and a medium effect size for the utility score. Our result is similar to the finding found in a meta-analysis, which reported that cognitive behavioral therapy (CBT), one of the main foundations of Tele-SSM intervention, had a moderate size effect (Hedges’ g = 0.63) in improving HRQoL for adults with depression [19]. Moreover, the effect sizes of this intervention are greater than the effect sizes of a self-management program for people with depression which is a group-intervention grounded in social learning theory (a medium effect size for EQ-VAS and a small effect size for utility score) [47]. Compared to age-matched in the general population, there was no significant difference in EQ-VAS score between participants after completing the intervention and the general population.

Despite being improved after participating in the intervention, the proportions of problems in usual activities, pain/discomfort, and anxiety/depression dimensions were significantly higher as well as utility scores were still significantly lower in comparison to the age-matched general population. As demonstrated in previous studies [1, 17, 22], our findings extend these findings in that we also found a clear and consistent pattern as people with depression who had improvement at the end of intervention with better total health status still have a substantially lower HRQoL compared to the general population. These findings suggest the positive effects of the Tele-SSM intervention on HRQoL of people with depression; however, people with depression still need further support beyond the intervention to catch up with the general population.

We found an intercorrelation between depression, stress, and anxiety scores both at baseline and at the end of the intervention suggesting the comorbidities between depression and anxiety. The comorbidity between depression and anxiety was proven in a meta-analysis of longitudinal studies [23]. The relationship between stress and anxiety/depression was bidirectional [7, 38]. Stress and depression were associated with epigenetic changes in genes related to resilience and susceptibility to stress, including stress-response genes [38]. Stress could produce anxiety and anxiety can trigger another cycle of stress, while stress was also a major contributor to depression [5]. The association of depression and HRQoL is consistent with previous studies [8,11,20,40,44]. To explain the impact of depression on HRQoL, a research found that depressive symptoms are directly associated or indirectly associated with HRQoL through the mediation of impaired function [53].

Regarding the association between stress, anxiety, and HRQoL, we found that stress and anxiety were not associated with HRQoL controlling for depression symptoms. Our finding is consistent with the findings from another research which found that the association between anxiety and HRQoL disappeared when depression was included in the regression model [11]. However, this finding is different from the finding from the study of Brenes with 919 participants, the regression analysis found that both depression and anxiety were significantly related to lower HRQoL [3]. While the impact of anxiety on HRQoL still needs further investigation, the impact of depression on HRQoL seems concrete. Even controlling for demographic characteristics and other mental health problems including anxiety and stress, depression is still negatively related to HRQoL, which implies the loss in quality-adjusted life-years for individuals with depression as well as for their family, community and society at large.

### Limitations

Firstly, this was neither a randomized control trial nor a case–control study. Without a control group, the study results should be interpreted with caution as there might be other elements affecting the difference between pre-post intervention. Secondly, the study has a small sample size, with the majority being female (90%) and younger than 50 years old (95%). The main reason causing a small sample size of male could be gender norms that men should be strong, should not show weakness, could negatively affect the disclosure of mental health issues and mental health-seeking behaviors among men of mental health and/or depression among the Vietnamese population [51]. Reasons to explain the small number of 50 + years old participants could be their difficulties in using the technology. As a result, these challenges could limit the generalizability of our study for Vietnamese adults with depression. In addition, COVID-19 occurred during the study time which might affect the quality of life of the study participants compared with the quality of life of the general population which was examined before the COVID-19 pandemic. However, a study in Vietnam exploring the impact of the COVID-19 pandemic on general population, people in quarantine and people with self-isolation found that COVID-19 does not affect quality of life of the Vietnamese [50]. The reason could be that COVID-19 might be not a risk factor for quality of life itself, but how it affects the mental health of people in the pandemic. Therefore, the difference in the COVID-19 situation between the two studies might not affect the validity of the comparison that much. Finally, the comparison between the study sample and the general population sample was matched by ages only, there might be the differences in other characteristics might affect the comparison between the two samples.

## Conclusions

This study has been among the first studies in Vietnam describing the health profile of people with depression. Our findings indicate that HRQoL is significantly impaired in people with depression and significantly lower than the age-matched general population. Depression was associated with lower HRQoL controlling for stress and anxiety symptoms. These findings highlight the importance of tackling depression as depression consistently impairs HRQoL in the empirical evidence.Tele-SSM intervention improved the HRQoL of people with depression, however, HRQoL impairments were still persistently lower than the age-matched general population except for the EQ-VAS score. Beyond intervention period, people with depression may need further support to catch up with HRQoL of general population. The study provides initial insights on the impact of Tele-SSM intervention with HRQoL. Given limited resources settings, community-based care using mobile health is a high potential to improve the accessibility to care for people with depression. A randomized controlled trial of the Tele-SSM intervention should be conducted to evaluate the effectiveness and cost-effectiveness of the intervention.

## Data Availability

The quantitative data that support the findings of this study are openly available at https://bit.ly/49RBPuQ. The general population data of this study are available on request from https://bit.ly/4bn6UrD. The general population data are not publicly available due their containing information that could compromise the privacy of research participants.

## Abbreviations

CBT: Cognitive behavioral therapy
DASS-21: 21-Item Depression, Anxiety and Stress Scale
HRQoL: Health-related quality of life
ISDS: Institute for Social Development Studies
KAP: Knowledge-Attitude-Practice
NICE: The UK National Institute for Health and Care Excellence
PHQ-9: Patient Health Questionnaire
SSM: Supported self-management
